# Spiders fluoresce variably across many taxa

**DOI:** 10.1098/rsbl.2007.0016

**Published:** 2007-04-03

**Authors:** Kindra Andrews, Scott M Reed, Susan E Masta

**Affiliations:** 1 Department of Biology, Portland State UniversityPO Box 751, Portland, OR 97207, USA; 2 Department of Chemistry, Portland State UniversityPO Box 751, Portland, OR 97207, USA

**Keywords:** fluorescence, fluorophores, ultraviolet, Araneae, visual signalling

## Abstract

The evolution of fluorescence is largely unexplored, despite the newfound occurrence of this phenomenon in a variety of organisms. We document that spiders fluoresce under ultraviolet illumination, and find that the expression of this trait varies greatly among taxa in this species-rich group. All spiders we examined possess fluorophores in their haemolymph, but bright fluorescence appears to result when a spider sequesters fluorophores in its setae or cuticle. By sampling widely across spider taxa, we determine that fluorescent expression is labile and has evolved multiple times. Moreover, examination of the excitation and emission properties of extracted fluorophores reveals that spiders possess multiple fluorophores and that these differ among some families, indicating that novel fluorophores have evolved during spider diversification. Because many spiders fluoresce in wavelengths visible to their predators and prey (birds and insects), we propose that natural selection imposed by predator–prey interactions may drive the evolution of fluorescence in spiders.

## 1. Introduction

Fluorescence occurs when molecules called fluorophores absorb light at one wavelength and then emit light at a longer wavelength. In recent years, fluorescence has been described from a disparate array of living organisms (e.g. Mazel *et al*. 2004; Gandia-Herrero *et al*. 2005; Haddock *et al*. 2005). Still, we understand little about the taxonomic distribution, evolutionary history or function of this trait.

In corals, fluorophores are widely distributed taxonomically, and a variety of fluorescent proteins have evolved from a common ancestral protein (Labas *et al*. 2002; Ugalde *et al*. 2004). However, the possible functional role(s) of fluorescence is unknown. Likewise, all known species of scorpions have cuticles that fluoresce, suggesting that fluorescence may not play an ecological role (e.g. Fasel *et al*. 1997; Frost *et al*. 2001). In contrast, fluorescence is known from only a few types of birds (parrots) and crustaceans (mantis shrimp), but has been suggested to function as a visual signal in intraspecific communication in each of these organisms (Arnold *et al*. 2002; Mazel *et al*. 2004). Thus, fluorescence appears to be distributed haphazardly across the tree of life, but has the potential to function adaptively in at least some organisms.

We document that spiders, a species-rich and ecologically diverse group of organisms, possess fluorophores and can fluoresce. Remarkably, the externally visible expression of UV-induced fluorescence varies considerably both among portions of the body in individual spiders and from species to species. The expression of fluorescence appears to be controlled by sequestration of fluorophores in different regions of the body (figure 1*a*,*b*), suggesting that natural selection may act to control expression. We provide evidence that spiders are the only group known in which fluorescence is (i) taxonomically widespread, (ii) variably expressed, (iii) evolutionarily labile, and (iv) probably under selection and potentially of ecological importance for intraspecific and interspecific signalling.

## 2. Material and methods

### (a) Survey of fluorophore occurrence

To ascertain whether spiders in general possess fluorophores, we visually examined abdominal haemolymph in 13 spiders from 10 divergent families (table S1 in the electronic supplementary material) under a 302 nm ultraviolet (UV) lamp.

### (b) Fluorescence intensity analyses

Adult female spiders from 45 species, representing 41 genera and 19 families, were quantified for visible fluorophore expression with the use of a 302 nm UV lamp and a QImaging 3.3-megapixel digital CCD color camera connected to a Leica MZ9.5 stereomicroscope. Spiders were euthanized by freezing, pinned and then photographed at 15.2× magnification, first with white light, followed immediately by a 20.7 s exposure under 302 nm UV light in a darkroom. The images were imported into Image-Pro, and an image depicting only the intensity channel was created. Three replicate traces were made around the illuminated dorsal half of each spider's abdomen (the largest visible surface area of a spider) and pixel intensity was measured.

Since some spiders show distinct fluorescent patterns juxtaposed against a dark background, we classified spiders into dim, intermediate and bright intensity classes on the basis of the maximum intensity class (1–10) attained. Spiders with dorsal areas no brighter than class 4 were assigned to the dim intensity bin, spiders having maximal dorsal intensities in classes 5–7 were classified as intermediate and spiders whose brightest dorsal areas were in classes 8–10 were designated as bright. These three categories were used to map fluorescence intensity onto a morphological phylogeny of araneomorph spiders (Coddington *et al*. 2004), as shown in figure 1*c*.

### (c) Fluorescence excitation and emission spectra

We measured the fluorescent spectra of extractions from four species of spiders from four families: Araneidae (*Araneus diadematus*); Dysderidae (*Dysdera crocata*); Theridiidae (*Enoplognatha ovata*); and Thomisidae (*Misumena vatia*). Spider abdomens were ground in 95% ethanol and allowed to sit in the dark for 48 h at room temperature. Extractions were centrifuged at 14 000 r.p.m. for 5 min, and the supernatant was analysed on a steady-state PTI fluorimeter using 75 W arc lamp excitation. Spectra were recorded using a 2 nm bandpass on excitation and emission monochromators, a 1 nm data interval and an integration time of 0.1 s.

Our imaging system was sensitive to wavelengths of light emitted in the range visible to humans (approx. 400–750 nm), whereas our spectral analysis of fluorophores revealed that some peak emissions were below this range (peaks were 326–340 nm). However, because the tails of these emission peaks (figure 2) extended within the range captured by the imaging system, they were visible to us via image capture. Further evidence that the camera was able to detect these tails of the fluorescent spectra comes from the spider *E. ovata*, which fluoresced brightly with our imaging system, despite the fact that we found it possesses only a single fluorophore, with peak emission at 340 nm. We also confirmed that the 302 nm UV (lambda max=307 nm) light source we used for image capture was able to excite all fluorophores we found to be present in spiders; fluorimetric analyses showed that the excitation spectra of these fluorophores (peaking from 288 to 333 nm) all ranged across 302 nm.

## 3. Results

We document visible fluorescence from both the cuticle (figure 1*a*) and the setae (figure 1*b*) of some spiders. All 13 spiders we examined were found to possess fluorescent haemolymph, and all 45 taxa had eyes and joints that fluoresced, indicating that all possessed fluorophores. However, the externally visible expression of fluorescence in setae and cuticle varied markedly among species. Brightly fluorescent spiders were found in eight families, and spiders with intermediate fluorescence occurred in 11 families (figure 1*c*; table S1 in the electronic supplementary material). Of the 10 families for which we sampled multiple taxa, eight included species whose fluorescence was classified in different categories (figure 1*c*).

The excitation and emission spectra of the fluorophores extracted from the spiders revealed a diversity of fluorophores. Figure 2 illustrates representative emission spectra recorded in ethanol. Peak shape and lambda max did not vary with sample concentration or solvent polarity. Maximal excitation of the fluorophores was achieved with ultraviolet wavelengths (from 288 to 333 nm), indicating that fluorescence occurs primarily under ultraviolet irradiance. The peak emissions from these fluorophores ranged from the ultraviolet (325 nm) to the visible (466 nm) portions of the spectrum (figure 2). Samples from three spiders indicated at least two unique emission peaks per species (figure 2), suggesting the presence of multiple discrete fluorophores within species.

## 4. Discussion

The expression of fluorescence appears to be evolutionarily quite labile in spiders, varying both within and among families. The phylogenetic distribution of fluorescence intensity (figure 1*c*) reveals that evolutionary shifts in expression have occurred multiple times within araneomorph spiders. The current lack of resolution of the relationships among spider families and genera prevents exact calculation of the number of shifts in fluorescence intensity that have occurred during the diversification of spiders. However, the fact that there is variation among genera within single families indicates that there do not appear to be strong phylogenetic constraints on fluorescent expression.

The observed variation in fluorescence may result from several causes. Sequestration of fluorophores from haemolymph into the cuticle or setae intensifies fluorescence by increasing the amount of light reaching the fluorophores. A thick and opaque cuticle inhibits fluorescence by blocking light before it reaches the haemolymph, whereas a thin and transparent cuticle allows light to reach and excite fluorophores in the haemolymph. Finally, intensity may vary with the type and number of fluorophore(s) present.

Our fluorimetric analyses indicate that some spiders possess multiple fluorophores, and that novel fluorophores arose during the evolutionary history of spiders. Estimating the actual diversity of fluorophores across Araneae as a whole must await further research, but the variation found in our present sampling alone suggests that spiders as a group may possess a diversity of these chemicals.

The fact that fluorescence is both widespread and variable throughout this ecologically diverse group suggests that natural selection may be driving its expression. Spiders inhabit a wide range of environments, vary considerably in their life histories, and employ a multiplicity of foraging strategies and mating behaviours. Given such variation, we may expect that fluorescence could prove adaptive or maladaptive for a number of reasons, depending on species and context. Only some spiders appear to sequester fluorophores in their cuticles or setae so as to produce bright externally visible fluorescence. Moreover, fluorescence is often expressed in specific regions of the cuticle or in certain setae in ways that create novel patterns (figure 1*a*) or that highlight existing colour patterns (figure 1*b*) on the body surface. The ubiquity of fluorophores in haemolymph may suggest that they serve some vital metabolic or physiological function. However, their sequestration in externally visible portions of the body—in discrete patterns, and in some species but not others—hints at adaptive ecological functions.

In most organisms studied previously, the potential functions of fluorescence are not well understood. However, in a few organisms, fluorescence has been suggested to serve as a visual signal in communication among conspecifics (Arnold *et al*. 2002; Mazel *et al*. 2004; Lim *et al*. 2007). Most spiders have poor vision and rely primarily on vibratory and chemical cues to sense predators, prey and mates. Thus, we predict the evolution of visual signals for intraspecific communication to be limited in most spiders.

However, many prey (insects) and predators (birds and insects) of spiders possess acute visual abilities, generally extending into ultraviolet portions of the spectrum in which some spiders fluoresce most intensely. Therefore, it seems likely that insects and birds may exert selective pressures on the expression of fluorescence in spiders. Ultraviolet cues are important in spider predator and prey interactions (e.g. Craig & Bernard 1990; Chittka 2001; Heiling *et al*. 2003). Additionally, in the visually oriented jumping spiders, ultraviolet reflectance plays a role in intraspecific communication (Lim & Li 2006*a*,*b*). Fluorescence in the ultraviolet would have the same visual effect as reflectance in the ultraviolet—and indeed these can be difficult to distinguish. We suggest that the fluorescence we document may play a role similar to ultraviolet reflectance in serving to make spiders more cryptic to their predators and prey in certain ecological contexts, such as background-matching on fluorescent flowers (Gandia-Herrero *et al*. 2005). In other contexts, fluorescence could enhance conspicuousness for prey attraction or communication among spiders that are visually oriented. Selection could conceivably act in complex ways to enhance or inhibit the expression of fluorescence.

## Figures and Tables

**Figure 1:**
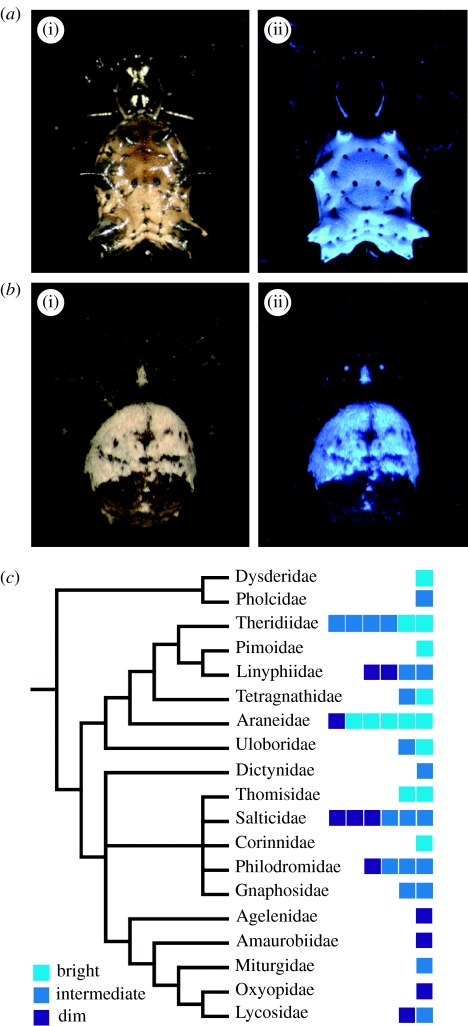
(*a*) *Micrathena gracilis* under white light (i) and UV illumination (ii). Note that entire cuticle of the abdomen fluoresces under UV, despite dark colouration of protuberances under white light. (*b*) *Hyptiotes* sp. under white light (i) and UV illumination (ii). Note that white setae on anterior abdomen fluoresce, whereas dark setae on posterior abdomen do not. (*c*) Distribution of visible fluorescence across spider families. Each square corresponds to one species sampled and colours denote intensity of fluorescence. Phylogenetic tree is simplified from Coddington *et al*. (2004).

**Figure 2:**
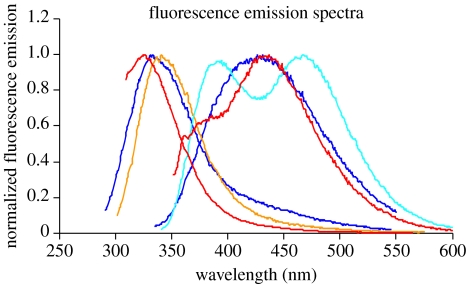
Normalized emission spectra of fluorophores from four spider species. *Araneus diadematus* (Araneidae) spectra are coloured red (288 and 330 nm excitation). *Dysdera crocata* (Dysderidae) spectra are coloured dark blue (290 and 328 nm excitation). *Enoplognatha ovata* (Theridiidae) shows only a single fluorophore peak (coloured gold), with excitation at 291 nm. *Misumena vatia* (Thomisidae) shows a bimodal spectrum (coloured light blue) with excitation at 333 nm, consistent with the presence of two different fluorophores with similar maximal excitations.

## Supplementary Material

Table S1 Familes, genera, and species of spiders used to quantify fluorescence intensity

## References

[bib1] Arnold K.E, Owens I.P.F, Marshall N.J (2002). Fluorescent signaling in parrots. Science.

[bib2] Chittka L (2001). Camouflage of predatory crab spiders on flowers and the colour perception of bees (Aranida: Thomisidae/Hymenoptera: Apidae). Entomologia Generalis.

[bib3] Coddington J.A, Giribet G, Harvey M.S, Prendini L, Walter D.E, Cracraft J, Donoghue M.J (2004). Arachnida. Assembling the tree of life.

[bib4] Craig C.L, Bernard G.D (1990). Insect attraction to ultraviolet-reflecting spider webs and web decorations. Ecology.

[bib5] Fasel A, Muller P.A, Suppan P, Vauthey E (1997). Photoluminescence of the African scorpion “*Pandinus imperator*”. J. Photochem. Photobiol. B.

[bib6] Frost L.M, Butler D.R, O'Dell B, Fet V, Fet V, Selden P.A (2001). A coumarin as a fluorescent compound in scorpion cuticle. Scorpions 2001. In Memoriam Gary A. Polis.

[bib7] Gandia-Herrero F, Garcia-Carmona F, Escribano J (2005). Floral fluorescence effect. Nature.

[bib8] Haddock S.H.D, Dunn C.W, Pugh P.R, Schnitzler C.E (2005). Bioluminescent and red-fluorescent lures in a deep-sea siphonophore. Science.

[bib9] Heiling A.M, Herberstein M.E, Chittka L (2003). Pollinator attraction: crab-spiders manipulate flower signals. Nature.

[bib10] Labas Y.A, Gurskaya N.G, Yanushevich Y.G, Fradkov A.F, Lukyanov K.A, Lukyanov S.A, Matz M.V (2002). Diversity and evolution of the green fluorescent protein family. Proc. Natl Acad. Sci. USA.

[bib11] Lim M.L, Li D (2006a). Behavioral evidence of UV sensitivity in jumping spiders (Araneae: Salticidae). J. Comp. Physiol. A.

[bib12] Lim M.L.M, Li D (2006b). Extreme ultraviolet sexual dimorphism in jumping spiders (Araneae: Salticidae). Biol. J. Linn. Soc.

[bib13] Lim M.L.M, Land M.F, Li D (2007). Sex-specific UV and fluorescence signals in jumping spiders. Science.

[bib14] Mazel C.H, Cronin T.W, Caldwell R.L, Marshall N.J (2004). Fluorescent enhancement of signaling in a mantis shrimp. Science.

[bib15] Ugalde J.A, Chang B.S.W, Matz M.V (2004). Evolution of coral pigments recreated. Science.

