# Supplementary material for: Spiders fluoresce variably across many taxa
Source: Biol Lett. 2007 Apr 3;3(3):265–7. doi: 10.1098/rsbl.2007.0016 (PMC2104643; doi:10.1098/rsbl.2007.0016)
Supplement: Supplementary file 1 [file rsbl20070016s10.doc]

**Table S1**. Families, genera, and species of spiders used to quantify visible fluorescence intensity. An asterisk indicates that fluorimetric data is presented in Figure 2.

| **Genus and species** | **Family** | **Fluorescence**  **Classification** | **Hemolymph**  **fluorescence** |
| --- | --- | --- | --- |
|  |  |  |  |
| *Hololena sp* | Agelenidae | dim | X |
| *Callobius sp.* | Amaurobiidae | dim |  |
| *Araneus diadematus** | Araneidae | bright | X |
| *Argiope aurantia* | Araneidae | bright | X |
| *Eustala anastera* | Araneidae | bright |  |
| *Larinioides patagiatus* | Araneidae | bright |  |
| *Micrathena gracilis* | Araneidae | bright |  |
| *Zygiella dispar* | Araneidae | dim | X |
| *Phurotimpus borealis* | Corinnidae | bright |  |
| *Cicurina sp.* | Dyctinidae | intermediate |  |
| *Dysdera crocata** | Dysderidae | bright | X |
| *Drassyllus insularis* | Gnaphosidae | intermediate |  |
| *Scotophaeus blackwalli* | Gnaphosidae | intermediate | X |
| *Bathyphantes chico* | Linyphiidae | dim |  |
| *Hypselistes florens* | Linyphiidae | dim |  |
| *Neriene sp.* | Linyphiidae | intermediate |  |
| *Pityohyphantes sp.* | Linyphiidae | intermediate |  |
| *Pirata sp.* | Lycosidae | intermediate |  |
| *Schizocosa sp.* | Lycosidae | dim |  |
| *Cheiracanthium inclusum* | Miturgidae | intermediate | X |
| *Oxyopes scalaris* | Oxyopidae | dim |  |
| *Philodromus alascensis* | Philodromidae | dim |  |
| *Philodromus dispar* | Philodromidae | intermediate |  |
| *Philodromus insperatus* | Philodromidae | intermediate |  |
| *Tibellus oblongus* | Philodromidae | intermediate |  |
| *Pholcus phalangioides* | Pholcidae | intermediate | X |
| *Pimoa altioculata* | Pimoidae | bright |  |
| *Phanias sp.* | Salticidae | dim |  |
| *Metaphidippus vitis* | Salticidae | dim |  |
| *Pelegrina sp.* | Salticidae | intermediate |  |
| *Phanias sp.* | Salticidae | dim |  |
| *Phidippus sp.* | Salticidae | intermediate | X |
| *Salticus scenicus* | Salticidae | intermediate |  |
| *Plesiometa argyra* | Tetragnathidae | bright |  |
| *Tetragnatha versicolor* | Tetragnathidae | intermediate |  |
| *Enoplognatha ovata** | Theridiidae | bright | X |
| *Lactrodectus hesperus* | Theridiidae | intermediate |  |
| *Steatoda sp.* | Theridiidae | bright | X |
| *Steatoda sp.* | Theridiidae | intermediate |  |
| *Steatoda sp.* | Theridiidae | intermediate |  |
| *Theridion sp.* | Theridiidae | intermediate |  |
| *Misumena vatia** | Thomisidae | bright | X |
| *Misumenops sp.* | Thomisidae | bright |  |
| *Philoponella sp.* | Uloboridae | intermediate | X |
| *Hyptiotes sp.* | Uloboridae | bright |  |
